# Contribution of Final-Year Medical Students to Hypertension Diagnosis in Primary Care Units

**DOI:** 10.3390/clinpract15110216

**Published:** 2025-11-20

**Authors:** Nikolaos Evangelidis, Areti Triantafyllou, Magda Gavana, Vasileios Gkolias, Styliani Ouzouni, Paschalis Evangelidis, Ilias Theodoropoulos, Despoina Symintiridou, Evangelia Naka, Ioannis Staikos, Martha Andreou, Stefanos Tsotoulidis, Stamatina Lamprou, Maria Dragasaki, Eirini Kada, Anna-Bettina Haidich, Michael Doumas, Emmanouil Smyrnakis

**Affiliations:** 1 Laboratory of Primary Health Care, General Practice and Health Services Research, School of Medicine, Aristotle University of Thessaloniki, 54124 Thessaloniki, Greece; evangeln@auth.gr (N.E.); magda.gavana@gmail.com (M.G.); vgkolia@auth.gr (V.G.); pascevan@auth.gr (P.E.); smyrnak@auth.gr (E.S.); 2 Primary Health Care Research Network, Aristotle University of Thessaloniki, 54124 Thessaloniki, Greece; souzouna@auth.gr (S.O.); eliasxr@yahoo.gr (I.T.); dsiminti@gmail.com (D.S.); g.staikos@gmail.com (I.S.); sttsot@yahoo.gr (S.T.); mariapaelas@gmail.com (M.D.); irenekada@yahoo.gr (E.K.); haidich@auth.gr (A.-B.H.); 3 First Propedeutic Department of Internal Medicine, AHEPA University Hospital, Aristotle University of Thessaloniki, 54636 Thessaloniki, Greece; 4 Second Propedeutic Department of Internal Medicine, Hippokration General Hospital, Aristotle University of Thessaloniki, 54642 Thessaloniki, Greece; doumasm@auth.gr; 5 Department of Hygiene, Social & Preventive Medicine and Medical Statistics, School of Medicine, Faculty of Health Sciences, Aristotle University of Thessaloniki, 54124 Thessaloniki, Greece

**Keywords:** hypertension, medical education, patient care, primary care

## Abstract

Background/Objective: Worldwide, ~45% of hypertensives remain undiagnosed, and ~26% are adequately controlled. The active involvement of all healthcare professionals in diagnosing hypertension at primary health care units (PHCUs) is linked to better blood pressure (BP) control. There is currently no research examining the potential role of senior medical students in the diagnosis of hypertension. This study aimed to evaluate the contribution of final-year medical students’ active participation in the diagnosis of hypertension. The study also examined the prevalence and control of hypertension among health service users in Greek PHCUs. Methods: This is a cross-sectional convenience sample study. During clinical placement in PHC, sixth-year medical students received systematic training and performed BP measurements, according to the guidelines, in private, well-organized spaces. Adult patients and visitors were enlisted for BP measurements. The BP readings were provided to the participants so they could discuss any concerns about their BP with their physician. Statistical analysis was performed with SPSS. Categorical variables are presented as frequencies. Continuous variables were assessed for normality and, based on their distribution, are expressed as mean ± standard deviation or median (interquartile range). Appropriate tests were performed for the comparisons across groups (chi-square for the categorical variables, and two-sample *t*-test or Mann–Whitney test for continuous variables). A *p*-value < 0.05 was considered statistically significant. Results: In the present study, 124 medical students performed BP measurements in 68 PHCUs. BP was measured in 704 individuals, aged 61 (IQR: 48.0–73.0) years old; 58.8% were female, 68.3% of whom were patients. The prevalence of hypertension was 56.7%. The control rate was 44.9% (BP < 140 and 90 mmHg among all hypertensives), and the control rate under treatment was 61.0% (BP < 140 and 90 mmHg among treated hypertensives). The involvement of medical students contributed positively, increasing the diagnosis of hypertension in individuals who might not have their BP measured in routine clinical practice. Ninety-nine newly diagnosed hypertensives were detected. Students identified 220 uncontrolled hypertensives and 112 uncontrolled under-treated patients, who were then referred to the consultant physicians. Conclusions: Students played a critical role in diagnosing hypertension and identifying newly diagnosed hypertensive patients. Embracing interprofessional care in the diagnosis and management of hypertension is essential for achieving better outcomes for our patients. Engaging medical students in BP measurements is a practical and feasible approach to improve hypertension diagnosis and control, taking into consideration the increased workload of PHC physicians. While this action has important medical education implications, the impact on the knowledge level of medical students was not evaluated. Limitations of this study include the assessment of BP in one visit without home BP measurements or a second visit, lack of follow-up of newly diagnosed hypertensives, and the low average number of BP measurements per student.

## 1. Introduction

Cardiovascular disease (CVD) is the leading cause of mortality and morbidity worldwide, while arterial hypertension is the most significant modifiable CVD risk factor [1,2]. The prevalence of hypertension worldwide exceeds 35% of adult individuals [3,4]. Additionally, ~45% of hypertensives are undiagnosed, and only in ~26% is adequate control of blood pressure (BP) levels achieved [5]. In studies conducted in Greece, the prevalence of hypertension has been estimated at ~40% [6]. Τhe control rate is ~46% among treated hypertensive individuals [6].

The pivotal role of primary healthcare (PHC) in the management of hypertensive patients (from diagnosis to treatment) has been recognized and endorsed by the European Society of Hypertension (ESH) [7]. Performance of BP measurements is suggested during each patient visit at the PHC unit (PHCU) [8,9]. Nevertheless, regular measurements have not been established in all patients. Previous studies have shown that, in everyday clinical practice, the majority of PHC physicians fail to measure BP in all patients, despite established recommendations [10,11,12]. Data from multiple countries confirm that routine BP measurement is not systematically implemented, reflecting a gap between guidelines and everyday clinical practice [10,11]. A recent study of our group in Greek PHCUs demonstrated that BP is not routinely measured by most physicians, and only 13.2% measure BP in all patients in real-world practice [13]. Additionally, a significant number of hypertensive patients do not seek medical consultation and BP measurement because they are frequently asymptomatic and consider themselves healthy [14]. Thus, hypertension diagnosis is often delayed or missed.

BP measurements can be implemented not only by physicians but also by trained healthcare professionals [15]. It is also supported that when standardized protocols are followed, BP measurements performed by trained healthcare professionals are considered reliable [16]. Team-based management and interprofessional care (IPC) of hypertension is also supported by the Centers for Disease Control and Prevention and has significant benefits for the patients [15,17]. It should be noted that IPC refers to collaborative patient care, where different healthcare professionals contribute to patient assessment and management [18,19,20].

Several educational programs developed in the past have underlined the importance of medical students’ training at PHCUs, with significant benefits for the trainees [21,22]. Moreover, the role of medical students in public health promotion is undeniable. Examples of their contribution include their participation in actions to raise awareness, educating communities, and actively participating in health initiatives [21]. Educational programs have assessed medical students’ involvement in the diagnosis of chronic conditions. Through these projects, substantial benefits for both students and patients are highlighted [23,24]. Involving medical students in BP measurements could provide an opportunity to measure BP in patients and other PHCU users who are often overlooked. Additionally, based on the concept of IPC, the supervised involvement of medical students in BP measurement could be considered as a component of IPC. However, a significant number of medical students have a lack of knowledge of the accurate measurement technique of BP [25,26]. This emphasizes the need for focused training on BP measurement.

This study aimed to assess the contribution of final-year medical students in the diagnosis of hypertension. Moreover, the prevalence and control of hypertension were evaluated among adult visitors (patients, their accompanying persons, and other visitors) at PHCUs in Greece. Student contribution was defined as the measurement of BP after training to identify new or uncontrolled hypertensive patients and refer them to PHC physicians. Unlike previous studies focused mainly on medical students’ knowledge [25,26], this study combines hands-on practice with systematic training to increase the diagnosis of hypertension in PHCUs. To the best of our knowledge, this is the first study to present the role and potential of medical students’ participation in hypertension diagnosis.

## 2. Materials and Methods

### 2.1. Study Settings, Training, and Participation of Medical Students

**Figure 1:**
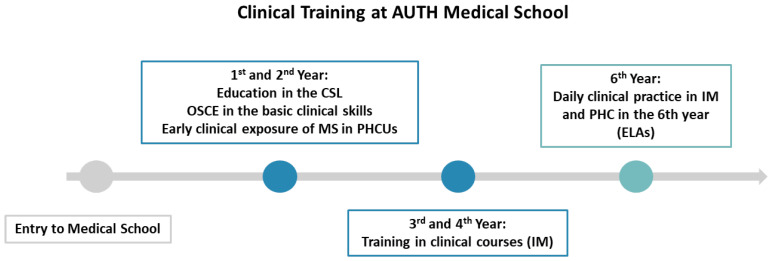
The figure illustrates the clinical education at AUTH Medical School. AUTH: Aristotle University of Thessaloniki, CSL: Clinical skills lab, ELAs: Experiential learning activities, MS: Medical students, IM: Internal medicine, OSCE: Objective structured clinical examination, PHC: Primary healthcare, PHCUs: Primary healthcare units.

In Greece, final-year medical students perform structured clinical placements in multiple medical specialties, including internal medicine, surgery, pediatrics, neurology, psychiatry, obstetrics, and gynecology, and PHC and general practice (GP). During their placement at PHCUs, students actively participate in patient care under the supervision of PHC physicians. By the final year, students are expected to perform accurate BP measurements in accordance with guidelines [27,28]. In Figure 1, the timeline of clinical training in Greece, and particularly at the medical school of Aristotle University of Thessaloniki (AUTH), is graphically presented. Most students at our school declared that they could independently perform BP measurements and evaluate home BP recordings upon their graduation [28]. Additionally, as presented in Figure 1, students at AUTH receive systematic training in the BP measurement technique in the first year of their studies and are evaluated with an objective structured clinical examination (OSCE).

During their clinical placement in PHC and GP, final-year medical students of AUTH participated in experiential learning activities (ELAs). Students were informed of all the available practice topics. The ELA’s topics concerned health promotion and prevention in the community. A training course on BP measurement and hypertension diagnosis in PHCUs was held for ELA participants. The training included the basic principles of BP measurement according to the ESH 2023 guidelines [29]. Additionally, all the participants were instructed on the basic principles of physician-patient communication. In our school, medical students received systematic training in BP measurements in the first semester of their studies and undertook an OSCE on basic clinical skills, including BP measurement. Moreover, students received further education on BP measurement and on hypertension diagnosis and management during the3ir previous 10-week clinical clerkship in their clinical training in internal medicine. Online support was available throughout the whole ELA period to clarify any queries. Four different cohorts of medical students from two academic years (2023–2024, 2024–2025) participated in this ELA. All medical students performed at least five BP measurements during their participation in the ELA. It should be noted that, to participate, students were required to perform a minimum of five BP measurements.

In each PHCU, an attending physician assisted students in organizing a private space for the BP measurements. Each student checked the validity of the BP monitor that was available in the PHCU according to STRIDE BP, an international scientific non-profit organization founded by hypertension experts with the mission of improving the accuracy of BP measurement and the diagnosis and management of hypertension globally [30]. The measurements were performed during one of the four weeks of their clinical placement in PHCUs. Students performed BP measurements under the supervision of an experienced attending physician.

Specific guides were given to all students. Students were instructed to provide the participants with their printed BP measurements. If a participant had significantly high BP [systolic BP (SBP) > 180 mmHg and/or diastolic BP (DBP) > 120 mmHg] or low BP (SBP < 90 mmHg and/or DBP < 60 mmHg), the on-call physician of the PHCU was immediately informed. All other individuals with SBP > 140 mmHg and/or DBP > 90 mmHg were referred to consult their physician, if applicable, or assigned to the physician at the PHCU on a regular appointment. Short anonymous reports were collected from all participants after the completion of the ELA. The ethics committee of the Medical School AUTH approved the study (protocol code: 91/2024), and the study was initiated in January 2024.

### 2.2. Study Design, BP Measurements, and Data Collection

This is a cross-sectional convenience sample study. Convenience sampling was applied, as data were collected from individuals who visited the PHCUs during the study period. The application of convenience sampling was chosen to reflect the “real” flow (“real world”) of patients and visitors [31]. The sample size reflected all eligible participants during the study period and was based on the minimum measurements that were obtained from each medical student (a minimum of 5, as will be analyzed in the next paragraphs). To minimize selection bias, all final-year students received standardized BP measurement training and conducted measurements under PHC physician supervision in private clinical spaces, as mentioned above.

BP measurements were performed in private spaces of the PHCUs. Adult volunteers (≥18 years old) were recruited for the measurements. Both patients attending for a regular or emergency visit at the PHCU and visitors (including patient accompanying persons and individuals visiting the PHCU for non-medical reasons) were invited to participate. Both patients and visitors were consecutively approached by medical students while they waited in the waiting area of the PHCU. Medical students explained the procedure of the study to the participants. Informed consent was obtained from all participants, including patients, accompanying persons, and visitors, for a non-medical reason. The exclusion criteria of this study included participants who were younger than 18 years old, patients in a life-threatening emergency status, or individuals who did not provide consent to participate in the study. The recruitment continued until each student completed the required minimum of five cases. However, students were permitted to recruit more individuals in the study beyond the minimum requirement at their discretion.

A short medical history was taken by the student. The presence of diabetes, dyslipidemia, and atrial fibrillation was recorded (self-reported by patients). Three BP measurements were performed in each patient at one-minute intervals. The estimated time the participant abstained from smoking was recorded (for current smokers). The values of SBP, DBP, and heart rate, and medical history were recorded in an online form created on Google Forms (Google, Mountain View, CA, USA). The average of the last two readings was calculated and used for the analysis. The following definitions were used for the diagnosis of hypertension and classification of the participants:

- Prevalence of Hypertension: SBP > 140 and/or/DBP > 90 mmHg and/or self-reported use of anti-hypertensive medication.
- Control: SBP < 140 and DBP < 90 mmHg among hypertensive individuals.
- Control under treatment: SBP < 140 and DBP < 90 mmHg in patients receiving anti-hypertensive medication.
- Normal BP (normotensives): SBP < 140 and DBP < 90 mmHg in individuals with no use of anti-hypertensive medication (self-reported).

The anti-hypertensive treatment was based mainly on the patient’s report, as well as on available health records.

### 2.3. Statistical Analysis

Descriptive statistics of categorical variables are presented as frequencies and percentages (%). Continuous variables were assessed for normality with the Kolmogorov–Smirnov test or Shapiro–Wilk test, as appropriate. Normally distributed parameters are expressed as mean ± standard deviation (SD) and non-normal as median and interquartile range (IQR). The IQR was presented as the 25th (Q1) and 75th (Q3) percentiles of the distribution. Comparisons between categorical variables (e.g., prevalence of hypertension by sex, dyslipidemia and diabetes status, and age groups) were conducted using the chi-squared test. The two-sample *t*-test or the Mann–Whitney test was used for the comparison of normally or non-normally distributed continuous variables, respectively (e.g., age between hypertensive and normotensive groups). A *p*-value of less than 0.05 was considered statistically significant. Statistical analysis was performed using SPSS. 28.0 statistical package (IBM SPSS Statistics for Windows, Version 28.0. IBM Corp, Armonk, NY, USA).

## 3. Results

### 3.1. Study Participants

One hundred twenty-four sixth-year medical students performed BP measurements in 68 PHCUs in all seven health territories of Greece. A total of 704 patients, accompanying persons, or visitors to PHCUs were recruited for the BP measurements. The demographic data of the study’s participants are presented in Table 1.

### 3.2. Prevalence and Control of Hypertension Among Participants

The prevalence of hypertension was found to be 56.7%. Table 2 presents the characteristics of the normotensives and hypertensive individuals. Prevalence was higher in men than women [69.0% versus (vs.) 48.1%, *p* < 0.001]. Additionally, patients with dyslipidemia (75.3% vs. 47.1%, *p* < 0.001) and diabetes mellitus (80.0% vs. 51.9%, *p* < 0.001) demonstrated a higher prevalence of hypertension. Individuals older than 80 years old were more likely to be hypertensives compared to younger individuals (88.1% vs. 52.5%, *p* < 0.001).

**Figure 2:**
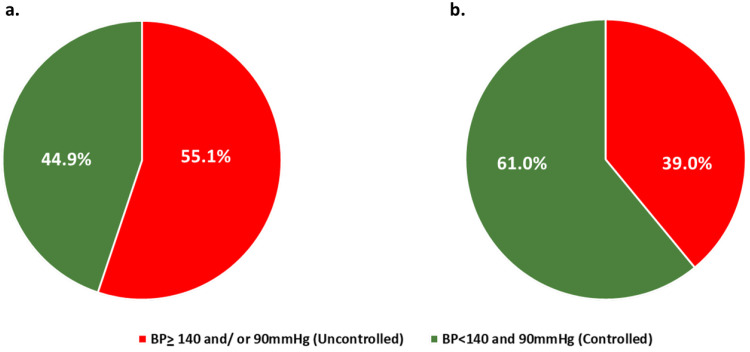
(**a**): Hypertension control among all hypertensives. (**b**): Control under treatment. BP: Blood pressure.

The control of hypertension among all hypertensive individuals (control) and patients under hypertensive medication (control under treatment) is presented in Figure 2. In Table 3, the characteristics of controlled and uncontrolled individuals under treatment are presented.

### 3.3. Contribution of Medical Students: Identification of Newly Diagnosed and Uncontrolled Hypertensive Individuals

**Figure 3:**
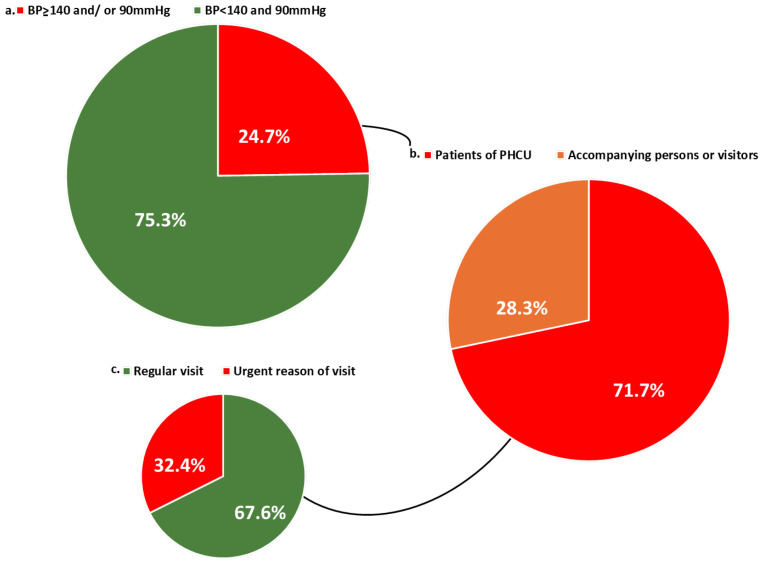
(**a**) Prevalence of hypertension among individuals not receiving antihypertensive medication. (**b**) Type of visitor among newly diagnosed hypertensive individuals (patient vs. accompanying person/visitor). (**c**) Reasons for PHCU visits among newly diagnosed hypertensive patients. BP: blood pressure, PHCU: Primary healthcare units.

Among the participants that medical students measured, 400 individuals did not have any diagnosis of hypertension and did not receive any anti-hypertensive medication. Between those, about ¼ (99) were diagnosed with hypertension according to the students’ measurements. Newly diagnosed hypertensives were both patients and accompanying persons/visitors, as presented in Figure 3. More specifically, BP was measured in 223 accompanying persons/visitors of PHCUs, of whom 21.1% (47/223) had BP > 140 and/or 90 mmHg, and 59.6% (28/47) of those did not receive anti-hypertensive medication (newly diagnosed). Prevalence of hypertension among accompanying persons/visitors was 39.5% (88/223), of which 36.4% (32/88) were controlled, and 67.2% (39/58) were controlled under treatment.

## 4. Discussion

In our study, final-year medical students took part in BP measurements during their clinical placement in PHC and GP settings. We propose a practical and structured plan for integrating medical students into BP measurements at PHCUs. Students measured BP of 704 individuals who visited PHCUs, and among them identified 99 individuals with newly diagnosed hypertension and 112 individuals whose BP was not adequately controlled despite receiving anti-hypertensive medication. All these participants were referred to their personal physician or to a physician at the PHCU. Additionally, 223 accompanying persons or visitors for non-medical reasons participated in BP measurements: 28 newly diagnosed hypertensives were identified, and 19 participants were uncontrolled despite receiving anti-hypertensives (19 of the total 58 visitors who received treatment). Considering that, due to the lack of time and personnel, BP is unfortunately not routinely measured in PHCUs, students’ contribution can be regarded as valuable, particularly in measuring the BP of individuals who visit the units as non-patients and would likely not have their BP checked otherwise [10,13]. The participation of medical students could potentially help overcome key obstacles in PHC, including high physician workload and limited time available with each patient [13]. By performing BP measurements, students can potentially screen for newly diagnosed or uncontrolled hypertensive patients and refer them to the PHC physician, which is particularly valuable given that BP is measured in only a small proportion of PHC patients (~13 to 30%) [10,13]. It should be noted that in our study, we did not compare diagnosis rates before vs. after students’ contribution, but rather aimed to demonstrate that medical students can contribute to the identification of hypertensive individuals who may not otherwise have had their BP measured in routine practice.

### 4.1. Comparison with Similar International and National Studies

The prevalence of hypertension in this study was 56.7%. In our sample, the control of hypertension was 45%, and the control under treatment was 61%. In PURE, a large cross-sectional, population-based study from 17 countries, the prevalence of hypertension was 40.8% and 32.5% of treated individuals had their BP controlled [32]. The differences in control rates between our study and PURE could potentially be attributed to the mixed-income countries that were included in this study, as in lower-income countries, lower control rates were reported [32]. The findings from the May Measurement Month (MMM) screening campaign for hypertension (60 countries, 715,518 participants) reported a prevalence of 36.0% and a control under treatment rate of 52.9% [5]. In agreement with our findings, 58.3% of treated hypertensives in the IBERICAN—a study conducted at PHCUs in Spain with 8066 participants—had their BP controlled [33]. Results from a screening survey in Italy (1354 participants, mean age: 56.3 ± 15.3 years) reported that 46.5% of hypertensives were controlled, in accordance with our findings [34].

In other population studies in Greece, such as the EMENO study, the prevalence of hypertension was estimated to be 39.6% among 4699 individuals aged ≥18 years old from the general population who participated in screening for hypertension [6]. Screening projects developed by the Greek national hypertension association in the context of MMM presented a prevalence of hypertension of 41.6 to 42.6% [35]. In a study developed by Triantafyllou et al. in a population consisting of elderly individuals in Greece, the prevalence of hypertension was 89% (mean age 74.3 ± 5.8) [36]. In the NEMEA study (mean age of participants 73.5 ± 6.1 years), the overall prevalence of hypertension was 69.1% [37]. The HYPERTENSHELL study presented a prevalence of 31.1% among 11,540 participants, and was higher (65.4%) in the elderly [38]. The relatively high prevalence in our population could be due to the high median age of the participants (61 years). Similar findings regarding hypertension control have arisen from other large screening studies [6,35]. Specifically, in the EMENO study, 45.9% of participants under anti-hypertensive medication were controlled [6]. While our findings broadly align with previous epidemiological studies, they primarily reflect the epidemiology of hypertension among PHCUs’ visitors and do not ultimately reflect the prevalence and control of hypertension in the general population. In this study, we primarily intended to demonstrate the meaningful contribution of medical students in the identification of both newly diagnosed and uncontrolled hypertensives, while the assessment and discussion of the prevalence and control of hypertension were only secondary.

### 4.2. Educational and Clinical Implications of Involving Students in Screening

According to previous studies, undergraduate medical students’ participation in health promotion and prevention is arguably beneficial for patients, the healthcare system, and students’ education [39,40]. The study by Schefer et al. was conducted in Germany at a clinical education ward, and final-year medical students actively participated in patient care under supervision [39]. This study reported that active student participation can have a positive impact not only on the learning process, but also on patient care outcomes and patient–physician interaction [39]. While the publication of Yardley et al. provides the theoretical foundations of ELA in medical education, it also outlines the basic principles of implementation of experiential learning in medical education [40]. Involvement of medical students in community education programs has been supported by medical students themselves, who report improved awareness of community health and increased confidence in their soft skills [41,42].

IPC has been proposed as a valuable tool for the management of hypertensive patients [13]. In a systematic review and meta-analysis of 25 studies, IPC practices were substantiated as beneficial for patients with hypertension in PHC (reduction in SBP and DBP). Regarding the decrease in SBP, the standard mean difference (SMD) was −0.31 [95% confidence interval (CI), −0.46 to −0.17, *p* < 0.001], and for DBP −0.28 (95% CI: −0.42–−0.14, *p* < 0.001) [15]. Bray et al. conducted a prospective cohort study (PCT) in 727 diabetic adults, evaluating the impact of IPC on the levels of BP. Findings suggested that SBP < 140 mmHg was more prevalent in the intervention group (69% vs. 57%, *p* < 0.01) [43]. Additionally, other PCTs suggest improved outcomes in SBP and DBP levels for patients with comorbidities such as chronic kidney disease and diabetes [44,45]. The RAMP-HT study presented that IPC management of hypertension contributed to better control of BP [45]. Previous studies have demonstrated the benefits of IPC in the management of hypertension. Our study differs in that it focuses specifically on the contribution of final-year medical students to detecting newly diagnosed and uncontrolled hypertensive patients within PHCUs.

The involvement of medical students in BP measurements could potentially free up valuable clinical time for PHC physicians, internal medicine physicians, or cardiologists. This additional time could be used to perform a more comprehensive assessment of target organ damage in hypertensive individuals. Moreover, advanced echocardiographic imaging techniques -such as speckle tracking echocardiography- to detect subclinical myocardial dysfunction in asymptomatic hypertensive patients could be performed, as suggested by recent studies [46,47].

Our team has, for several years, established a four-week medical attachment at PHCUs during the GP and PHC course in the final year of medical school [27]. Based on the previously published qualitative analysis of 44 participants of this ELA, participation offers significant benefits not only for patients but also for students and healthcare systems, as medical students reported that they gained knowledge on proper BP measurement and communication skills, as well. This ELA was developed and conducted in PHCUs all over Greece. From the qualitative analysis of the reports of some participants, it was concluded that the PHCUs’ environment was convenient for the implementation of the BP measurements [48]. Additionally, the strategic planning and the voluntary participation of the staff at the PHCUs were the main determinants for successfully carrying out this ELA [48]. Moreover, based on our previous experience from our students’ clinical practice at PHCUs, the support of the staff (GPs, nurses, and other healthcare professionals on a volunteer basis) through a large network of colleagues contributes positively to the implementation of our students’ clinical placements [27].

### 4.3. Limitations and Future Research Directions

A limitation of this study is that the participants were visitors and patients of PHCUs, meaning the prevalence of hypertension may not be representative of the general population. However, the estimation of the epidemiology of hypertension was a minor secondary aim of this study. However, PHC generally covers a representative part of the population, and in our research, we included patients from 68 PHCUs across all regions of Greece, supporting the representativeness of our sample. A limitation that should not be disregarded is that participants did not receive home BP measurements, and those with elevated BP were not reassessed at a second visit. Additionally, the hypertension diagnosis was based only on office BP measurements in one visit and self-referred use of anti-hypertensive medication. Another limitation of this study is that the medical students were not involved in the follow-up care of newly diagnosed and uncontrolled hypertensive patients.

In this study, we did not evaluate the impact of this ELA on participants’ knowledge levels or other educational outcomes. Another limitation of our study is that the average number of BP measurements per student was relatively low (~6 for each medical student). Although medical students received systematic theoretical training, had substantial clinical experience, and were under the supervision of an experienced physician, BP measurements were not compared with those taken by supervisors. However, in other large hypertension trials (e.g., SPRINT), BP measurements were performed by trained personnel using standardized measurement protocols without comparison to measurements obtained by supervising physicians [49,50].

Home BP measurements and follow-up patient visits can be incorporated into this ELA when it is conducted with future student cohorts. Additionally, in the future, the impact of this ELA on knowledge regarding hypertension could be assessed using structured questionnaires for all participants, before and after the participation.

## 5. Conclusions

This study presents, for the first time, the involvement of final-year medical students in organized BP measurements at PHCUs. The students’ contribution can be considered beneficial, as they measured 704 individuals and identified 99 new potential cases of hypertension, with more than 30% of these individuals not having visited the PHCU as patients. The participation of medical students in screening programs may help overcome challenges in clinical practice and improve the diagnosis of hypertension in the community. Students also gain valuable experience and develop their knowledge, clinical skills, and communication abilities. However, larger and prospective studies are needed to further evaluate the role of medical students in the management and support of hypertensive patients.

## Figures and Tables

**Table 1:** Characteristics of the study’s participants (N = 704).

Age (years), median (IQR)	61.0 (48.0–73.0)
Gender, N (%):	
Male	290 (41.2)
Female	414 (58.8)
Participant categories, N (%):	
Patients	481 (68.3)
Accompanying persons/visitors of PHCU	223 (31.7)
Reason for visiting the PHCU *, N (%):	
Regular visit	543 (77.3)
Emergency	148 (22.7)
Current smoker, N (%)	174 (24.7)
Under antihypertensive medication **, N (%):	
Yes	287 (41.2)
No	400 (57.5)
Don’t know	9 (1.3)
Dyslipidemia, N (%)	239 (33.9)
Diabetes mellitus, N (%)	120 (17.0)
Atrial fibrillation, N (%)	44 (6.3)

IQR: Interquartile range, PHCU: Primary healthcare unit. * 13 Participants had missing values (available: 691/704) on the reason for visiting the PHCU. ** 8 Participants had missing values (available: 696/704) on antihypertensive medication.

**Table 2:** Characteristics of the normotensive and hypertensive individuals.

	Normotensive Individuals N = 305	Hypertensive Individuals N = 399	*p*
Age (years), median (IQR)	50 (34–60.5)	69 (58–77)	<0.001
Gender, N (%):			
Male	90 (29.5)	200 (50.1)	<0.001
Female	215 (70.5)	199 (49.9)	
Category of participants, N (%):			
Patients	170 (55.7)	311 (77.9)	<0.001
Accompanying persons/visitors of PHCU	135 (44.3)	88 (22.1)	
Reason for visiting the PHCU, N (%):			
Regular visit	228 (77.6)	315 (79.3)	0.570
Emergency	66 (22.4)	82 (20.7)	
Current smoker, N (%)	81 (26.6)	93 (23.3)	0.322
Dyslipidemia, N (%)	59 (19.3)	180 (45.1)	<0.001
Diabetes mellitus, N (%)	24 (7.9)	96 (24.1)	<0.001
Atrial fibrillation, N (%)	4 (1.3)	40 (10)	<0.001

IQR: Interquartile range, PHCU: Primary healthcare unit.

**Table 3:** Characteristics of the controlled and uncontrolled individuals under treatment.

	Controlled Individuals Under Treatment N = 175	Uncontrolled Individuals Under Treatment N = 112	*p*
Age (years), median (IQR)	71 (62–79)	74 (66–79)	0.095
Gender, N (%):			
Male	79 (45.1)	63 (56.2)	0.066
Female	96 (54.9)	49 (43.8)	
Category of participants, N (%):			
Patients	136 (77.7)	93 (83.0)	0.273
Accompanying persons/visitors of PHCU	39 (22.3)	19 (17.0)	
Reason for visiting the PHCU, N (%):			
Regular visit	147 (85.0)	88 (78.6)	0.165
Emergency	26 (15.0)	24 (21.4)	
Current smoker, N (%)	37 (21.1)	19 (17.0)	0.384
Dyslipidemia, N (%)	85 (48.6)	66 (58.9)	0.086
Diabetes mellitus, N (%)	48 (27.4)	35 (31.3)	0.486
Atrial fibrillation, N (%)	21 (12.0)	15 (13.4)	0.728

IQR: Interquartile range, PHCU: Primary healthcare unit.

## Data Availability

The data presented in this study are available on request from the corresponding author.

## Abbreviations

The following abbreviations are used in this manuscript:

AUTH	Aristotle University of Thessaloniki
BP	Blood pressure
CI	Confidence interval
CVD	Cardiovascular disease
DBP	Diastolic blood pressure
ELAs	Experiential learning activities
ESH	European Society of Hypertension
IPC	Interprofessional care
IQR	Interquartile range
GP	General practice
MMM	May Measurement Month
OSCE	Observed structured clinical exams
PHC	Primary healthcare
PHCUs	Primary health care units
SBP	Systolic blood pressure
SMD	Standard mean difference
SD	Standard deviation
Vs.	Versus
